# Therapeutic Notes

**Published:** 1900-10-20

**Authors:** 


### THERAPEUTIC NOTES.

### TREATMENT OF BALDNESS.

Gessner (*Therap. Gazette*) advises early treatment of chronic seborrhœa by washing with tincture of soap and very hot water, followed by cold affusions. If there is eczema or irritation he uses the following ointment:

℞ Icthyol ♏xii.

Zinci oxidi,

Amyli āā ʒi.

Petrolati ad ℥i. M.

The loose hairs should be removed and the washing carried out once or twice a week, a little oil being applied afterwards. When the scalp is thoroughly clean he advises

℞ Sulphuris precip, gr. xx.—l.

Resorcini gr. x.—xxiv.

Acid. salicyl. gr. v.—xij.

Tr. Benzoini ♏xii.

Petrolati ℥i. M.

S. To be well rubbed into the scalp at night and the head covered by a cap.

If baldness already exists, he applies every night to the scalp by a stiff brush an ointment containing 5 or 10 per cent. of Chrysarobin, and employs the Faradic current daily with a brush.—*Practitioner*, February.

### THE ACTION OF DIGITALIS.

Sir T. Lauder Brunton, in a report to the International Congress,[1] supports the view that digitalis not only increases the cardiac contractions but also acts on the vessels both directly and through the vasomotor centre. This action even influences the pulmonary vessels, though to a less extent than those of the general circulation. The chief components of the drug are digitoxin, digitalin, digitalein, digitonin, and digitin. Their action is alike in kind but different in degree, digitoxin being by far the strongest. It is the chief component of Nativelle's "digitalin." Hence the great power of that preparation. Digitalis is useful in functional irregularities, except those due purely to dyspepsia. Secondly, in mitral incompetence, whether primary or when due to failure of compensation. In aortic regurgitation with full compensation digitalis may be harmful and favour syncope through the prolonged diastole. Similarly in fatty heart or very high tension it may have dangerous results unless we are careful to administer at the same time full doses of nitrites to lessen the resistance of the arterioles. In such cases, too, it is important to attend carefully to the condition of the bowels and of the liver.

In pneumonia Professor Huchard[2] advocates the use of digitalin in order to aid the heart on which the stress falls of maintaining the circulation through the congested lung. This assistance must be given early to prevent the dilating action of the backward pressure of blood before the heart muscle is weakened. By the increased diuresis, too, he considers that the elimination of the poison is quickened.

[1] Lancet, August 18th. [2] Medical Notes, May.

